# Adsorption of Cationic Peptides to Solid Surfaces of Glass and Plastic

**DOI:** 10.1371/journal.pone.0122419

**Published:** 2015-05-01

**Authors:** Kasper Kristensen, Jonas R. Henriksen, Thomas L. Andresen

**Affiliations:** 1 Department of Micro- and Nanotechnology, DTU Nanotech, Technical University of Denmark, Kongens Lyngby, Denmark; 2 Department of Chemistry, DTU Chemistry, Technical University of Denmark, Kongens Lyngby, Denmark; 3 Center for Nanomedicine and Theranostics, Technical University of Denmark, Kongens Lyngby, Denmark; University of Helsinki, FINLAND

## Abstract

Cationic membrane-active peptides have been studied for years in the hope of developing them into novel types of therapeutics. In this article, we investigate an effect that might have significant experimental implications for investigators who wish to study these peptides, namely, that the peptides adsorb to solid surfaces of glass and plastic. Specifically, we use analytical HPLC to systematically quantify the adsorption of the three cationic membrane-active peptides mastoparan X, melittin, and magainin 2 to the walls of commonly used glass and plastic sample containers. Our results show that, at typical experimental peptide concentrations, 90% or more of the peptides might be lost from solution due to rapid adsorption to the walls of the sample containers. Thus, our results emphasize that investigators should always keep these adsorption effects in mind when designing and interpreting experiments on cationic membrane-active peptides. We conclude the article by discussing different strategies for reducing the experimental impact of these adsorption effects.

## Introduction

Cationic membrane-active peptides with antimicrobial [1–3] and/or cell-penetrating properties [4, 5] have been studied intensely for years with the goal of developing them into new types of therapeutics. In the endeavor of studying and understanding these peptides, numerous advanced experimental and theoretical methods have been employed, resulting in a wealth of scientific articles. However, a quite important piece of information is often put in a side note in these articles: cationic membrane-active peptides adsorb to the walls of glass and plastic sample containers [6–8]. In spite of the significant experimental implications of this issue, it has only seldom been considered in its own right, except in a small handful of papers [9–12].

In this article, we systematically quantify the adsorption of the three α-helical cationic membrane-active peptides mastoparan X, melittin, and magainin 2 to the walls of commonly used glass and plastic sample containers. This systematic documentation is performed by use of analytical HPLC, which also previously has been used to study adsorption of peptides to solid surfaces of glass and plastic [13]. Our results clearly demonstrate that interactions between cationic membrane-active peptides and the surfaces of glass and plastic sample containers represent an issue that should not be underestimated by experimental investigators. We conclude the article by discussing different strategies for minimizing the experimental impact of this issue.

## Materials and Methods

### Materials

POPC (1-palmitoyl-2-oleoyl-*sn*-glycero-3-phosphocholine) and POPG (1-palmitoyl-2-oleoyl-*sn*-glycero-3-[phospho-rac-(1-glycerol)], sodium salt) were purchased from Avanti Polar Lipids (Alabaster, AL). HEPES (N-(2-hydroxyethyl)piperazine-N’-(2-ethanesulfonic acid)) and the corresponding sodium salt, MeCN (acetonitrile), TFA (trifluoroacetic acid), and NaCl were purchased from Sigma-Aldrich (St. Louis, MO). Melittin (GIGAVLKVLTTGLPALISWIKRKRQQ-NH_2_) was purchased from Peptide 2.0 (Chantilly, VA), and mastoparan X (INWKGIAAMAKKLL-NH_2_) and magainin 2 (GIGKFLHSAKKFGKAFVGEIMNS) were purchased from GL Biochem (Shanghai, China). Mastoparan X was further purified by semi-preparative HPLC (Waters semi-preparative HPLC equipped with a Waters 600 pump & controller and a Waters 2489 UV/vis detector, Waters, Milford, MA). The identity of the peptides was confirmed by MALDI-TOF (Bruker Reflex IV MALDI-TOF spectrometer, Bruker, Billerica, MA).

HPLC autosampler vials (2 mL, dimensions 12 × 32 mm, borosilicate glass, product no. C4010-1), screw caps for HPLC autosampler vials (open top cap, polypropylene, product no. C4010-1A), and septa for screw caps (diameter 10 mm, PTFE/silicone, product no. C4010-40) were purchased from National Scientific (Rockwood, TN). Borosilicate glass vials (2 mL, dimensions 12 × 36 mm, cat. no. 150901) and limited volume inserts for HPLC autosampler vials (borosilicate glass, 300 μL, dimensions 6 × 30 mm, cat. no. 150820) were purchased from Brown Chromatography Supplies (Wertheim, Germany). Polypropylene tubes (safe-lock tubes, 2 mL, inner diameter 9 mm, order no. 0030 120.094), Protein LoBind tubes (safe-lock tubes, 2 mL, inner diameter 9 mm, order no. 0030 108.132), and pipette tips were purchased from Eppendorf (Hamburg, Germany). (The Protein LoBind tubes also consist of polypropylene, but throughout this article, we refer to them as Protein Lobind tubes to distinguish them from the standard polypropylene tubes.) Quartz glass cuvette (Suprasil, 1.5 mL, inner dimensions 4 × 10 mm, order no. 119004F-10-40) was purchased from Hellma (Müllheim, Germany). Magnetic stirring bar (PTFE-covered, 2 × 5 mm, cat. no. 442-0361) was purchased from VWR (Radnor, PA).

### LUV preparation and characterization

LUVs (large unilamellar lipid vesicles) were prepared as previously described [14]: POPC/POPG (3:1) solutions were prepared in chloroform/methanol (9:1). The organic solvent was removed under a gentle stream of nitrogen. The samples were subsequently kept in vacuum overnight to remove the residual solvent. The lipid films were hydrated in 10 mM HEPES, 100 mM NaCl, pH 7.4 buffer with vigorous vortexing every 5 min for a period of 30 min. The hydrated lipid suspensions were then subject to 5 freeze-thaw cycles by alternately placing the sample vials in an isopropanol/dry ice bath and a warm water bath. Next, the lipid suspensions were extruded 21 times through a 100 nm polycarbonate filter (Whatman, Maidstone, UK) using a mini-extruder (Avanti Polar Lipids) to form LUVs. The size of the LUVs was checked by dynamic light scattering (ZetaPALS, Brookhaven Instruments, Holtsville, NY). Phosphorus concentrations of the LUV solutions were determined using the method of Rouser et al. [15], albeit with slightly modified reagent concentrations. In the following, the stated LUV concentrations refer to the total concentration of lipids in the samples.

### Peptide stock solutions

Peptide stock solutions were prepared as previously described [14]: Peptide stock solutions were prepared in 10 mM HEPES, 100 mM NaCl, pH 7.4 buffer. To prevent loss of peptides due to adsorption to tube walls and/or pipette tips, the peptide stock solutions were handled in Protein LoBind tubes at a high concentration of at least 100 μM. The extinction coefficients of peptides at 220 nm were calculated to be 40100 cm^−1^M^−1^ for mastoparan X, 46700 cm^−1^M^−1^ for melittin, and 23900 cm^−1^M^−1^ for magainin 2 by correlating the peptide concentration determined by an Antek 8060 chemiluminescent nitrogen detector (PAC, Houston, TX) to the absorbance of the same peptide sample, determined by a NanoDrop 2000c spectrophotometer (NanoDrop Products, Wilmington, DE). Given these extinction coefficients, peptide concentrations of stock solutions were then always determined by recording the absorbance at 220 nm using the NanoDrop 2000c spectrophotometer.

### Sample preparation for analytical HPLC

All samples were handled in 10 mM HEPES, 100 mM NaCl, pH 7.4 buffer, except where otherwise stated.

#### Concentration standard curve measurements

The sample preparation protocol for the concentration standard curve measurements was as follows:

1. 200 μL peptide standard solutions of varying peptide concentration were prepared by adding varying volumes of a 100 μM peptide stock solution to varying volumes of buffer in limited volume inserts in HPLC autosampler vials.
2. The HPLC autosampler vials were capped using screw caps with septa and vigorously vortexed for a few seconds.
3. The screw caps with septa were removed from the HPLC autosampler vials, and 50 μL 5 mM POPC/POPG (3:1) LUV solution was added to each of the limited volume inserts in the vials. The final volume of the solutions in the limited volume inserts was then 250 μL and the final LUV concentration was 1 mM.
4. The HPLC autosampler vials were capped using the screw caps with septa and vigorously vortexed for a few seconds.
5. Each of the samples was measured by use of analytical HPLC.

This protocol was designed with the goal of minimizing the loss of peptide due to surface adsorption: In Step 1, peptide standard solutions were mixed by use of 100 μM peptide stock solutions; the purpose of using peptide stock solutions with such high concentrations was to saturate the walls of the pipette tips with peptide to minimize the relative loss of peptide during pipetting. In Step 3, POPC/POPG (3:1) LUVs were added to the peptide standard solutions; the purpose of this step was to promote the peptides to desorb from the walls of the limited volume inserts and instead partition onto the LUVs.

#### Adsorption experiments

The general sample preparation protocol for the adsorption experiments was as follows:

1. Peptide solutions were prepared in a given type of sample container, generally by adding varying volumes of a 100 μM peptide stock solution to varying volumes of buffer.
2. The peptide solutions were incubated in the given type of sample container under a given set of experimental conditions.
3. 200 μL of each of the peptide solutions was transferred by pipette to limited volume inserts in HPLC autosampler vials.
4. The HPLC autosampler vials were capped using screw caps with septa and vigorously vortexed for a few seconds.
5. The screw caps with septa were removed from the HPLC autosampler vials, and 50 μL POPC/POPG (3:1) LUV solution was added to each of the limited volume inserts in the vials. The final volume of the solutions in the limited volume inserts was then 250 μL and the final LUV concentration was 1 mM.
6. The HPLC autosampler vials were capped using the screw caps with septa and vigorously vortexed for a few seconds.
7. Each of the samples was measured by use of analytical HPLC.

In many aspects, the sample preparation protocol for the adsorption experiments is very similar to the sample preparation protocol for the concentration standard curve measurements: In Step 1 of both protocols, peptide solutions are mixed by the use of 100 μM peptide stock solutions to minimize the amount of peptide lost during pipetting. Furthermore, Steps 4–7 of the sample preparation protocol for the adsorption experiments are identical to Steps 2–5 of the sample preparation protocol for concentration standard curve measurements. Therefore, in a given adsorption experiment, any decrease in peptide concentration relative to the concentration standard curve measurements must have been due to loss of peptide in Steps 2 and 3 of the sample preparation protocol of that adsorption experiment.

The only steps of the sample preparation protocol that differed between individual adsorption experiments was Steps 1 and 2; the following sections describe these steps of the sample preparation protocols for the individual adsorption experiments presented in this article.

#### Peptide loss during 1 h incubation in sample containers: 220 μL solutions

1. 220 μL peptide solutions of 1, 2, 5, 10, or 20 μM were prepared by adding varying volumes of a 100 μM peptide stock solution to varying volumes of buffer in borosilicate glass vials, polypropylene tubes, or Protein LoBind tubes.
2. Immediately after addition of peptide, the solutions were vigorously vortexed for a few seconds and then incubated for 1 h.

#### Peptide loss during 1 h incubation in sample containers: 2 mL solutions

1. 2 mL 2 μM peptide solutions were prepared by adding 40 μL 100 μM peptide stock solution to 1960 μL buffer in borosilicate glass vials, polypropylene tubes, Protein LoBind tubes, or quartz glass cuvettes.
2. Immediately after addition of peptide, the solutions in the borosilicate glass vials, polypropylene tubes, and Protein LoBind tubes were vigorously vortexed for a few seconds and then incubated for 1 h, and the solutions in the quartz glass cuvettes were placed on a magnetic stirrer for 1 h for constant stirring by a magnetic bar.

#### Peptide loss during successive transfers between sample containers

1. 250 μL 5 μM peptide solutions were prepared by adding 12.5 μL 100 μM peptide stock solution to 237.5 μL buffer in borosilicate glass vials, polypropylene tubes, or Protein LoBind tubes.
2. Immediately after addition of peptide, the solutions were vigorously vortexed for a few seconds and then incubated for 1 h. Some of the solutions were then successively transferred to new sample containers of the same kind as the one in which those solutions had just been incubated. Immediately after each transfer step, the solutions were vigorously vortexed for a few seconds and then incubated for 1 h.

#### Effect of NaCl concentration on peptide loss

1. 220 μL 2 μM peptide solutions were prepared by adding 4.4 μL 100 μM peptide stock solution (in 10 mM HEPES, 100 mM NaCl, pH 7.4 buffer) to 215.6 μL buffer (10 mM HEPES, pH 7.4 with either 0, 100, or 150 mM NaCl) in borosilicate glass vials, polypropylene tubes, or Protein LoBind tubes. Thus, the NaCl concentrations of the prepared peptide solutions were 2, 100, or 149 mM.
2. Immediately after addition of peptide, the solutions were vigorously vortexed for a few seconds and then incubated for 1 h.

Here, it should be mentioned that we also prepared peptide standard solutions in 10 mM HEPES, pH 7.4 buffer with 2, 100, or 149 mM NaCl to test whether the accuracy of the HPLC method was affected by the altered NaCl concentration. These peptide standard solutions were prepared by adding 4 μL 100 μM peptide stock solution (in 10 mM HEPES, 100 mM NaCl, pH 7.4 buffer) to 196 μL buffer (10 mM HEPES, pH 7.4 with either 0, 100, or 150 mM NaCl) in limited volume inserts in HPLC autosampler vials to a final volume of 200 μL and a final peptide concentration of 2 μM. Then, these peptide standard solutions were handled according to the sample preparation protocol for the concentration standard curve measurements.

#### Adsorption and desorption kinetics of mastoparan X: surface adsorption kinetics in buffer

1. 220 μL 2 μM mastoparan X solutions were prepared by adding 4.4 μL 100 μM mastoparan X stock solution to 215.6 μL buffer in borosilicate glass vials or polypropylene tubes.
2. Immediately after addition of mastoparan X, the solutions were vigorously vortexed for a few seconds and then incubated for 10 s, 1 h, or 24 h.

#### Adsorption and desorption kinetics of mastoparan X: surface adsorption kinetics in 1 mM POPC/POPG (3:1) LUV solution

1. 215.6 μL 1.02 mM POPC/POPG (3:1) LUV solutions were prepared in borosilicate glass vials or polypropylene tubes. The solutions were vigorously vortexed, and subsequently 4.4 μL 100 μM mastoparan X stock solution was added to each of the solutions. The final volume of the solutions was 220 μL, the final LUV concentration was 1 mM, and the final mastoparan X concentration was 2 μM.
2. Immediately after addition of mastoparan X, the solutions were vigorously vortexed for a few seconds and then incubated for 10 s, 1 h, or 24 h.

#### Adsorption and desorption kinetics of mastoparan X: kinetics of desorption induced by 1 mM POPC/POPG (3:1) LUV

1. 210 μL 2.1 μM mastoparan X solutions were prepared by adding 4.4 μL 100 μM mastoparan X stock solution to 205.6 μL buffer in borosilicate glass vials or polypropylene tubes. Immediately after addition of mastoparan X, the solutions were vigorously vortexed for a few seconds and 10 μL 22 mM POPC/POPG (3:1) LUV solution was added to each of the solutions. The final volume of the solutions was then 220 μL, the final LUV concentration was 1 mM, and the final mastoparan X concentration was 2 μM.
2. Immediately after addition of LUV, the solutions were vigorously vortexed for a few seconds and then incubated for 10 s, 1 h, or 24 h.

### Analytical HPLC measurements

Analytical HPLC was performed on a Shimadzu LC-2010C integrated HPLC system equipped with a UV/vis detector (Shimadzu, Kyoto, Japan). The injection volume was 80 μL for samples of mastoparan X together with 1 mM POPC/POPG (3:1) LUV and 50 μL for samples of melittin or magainin 2 together with 1 mM POPC/POPG (3:1) LUV. We found that when larger volumes were injected, peptide peak areas were no longer linearly correlated to the peptide concentration. The flow rate of the system was 1 mL/min. Mobile phases were (A) water with 5% MeCN and 0.1% TFA and (B) MeCN with 0.1% TFA. Gradients were linear from 85% A to 0% A over 12 min. Peptides and lipids were separated on a XTerra RP8 (5 μm, 4.6 × 150 mm) column (Waters). UV absorbances were recorded at 220 nm. Peptide peak areas were determined by using the LC Postrun Analysis software.

## Results

### Concentration standard curves

**Fig 1:**
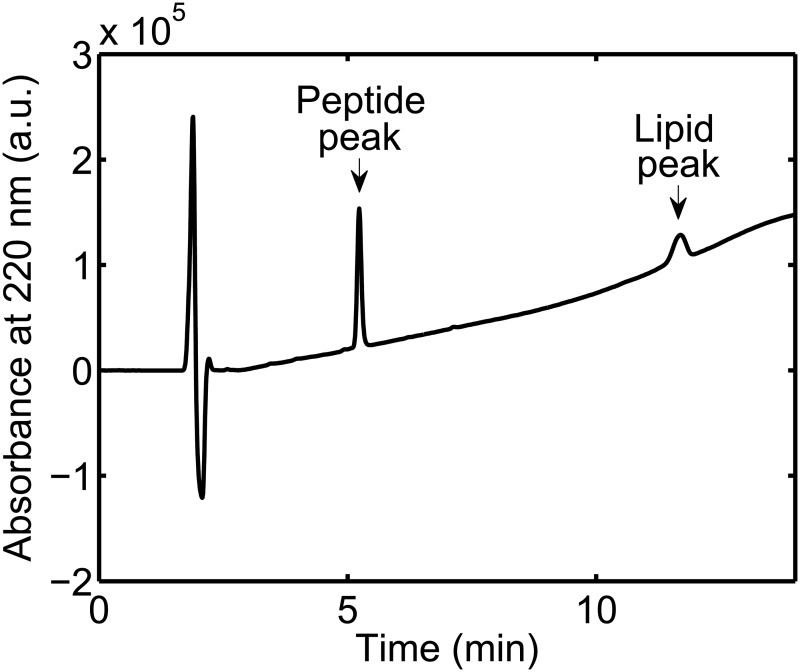
Example of HPLC chromatogram. The chromatogram was acquired from a 200 μL standard solution of 5 μM mastoparan X to which 50 μL 5 mM POPC/POPG (3:1) LUV had been added. Both a peptide peak and a lipid peak are visible in the chromatogram.

**Fig 2:**
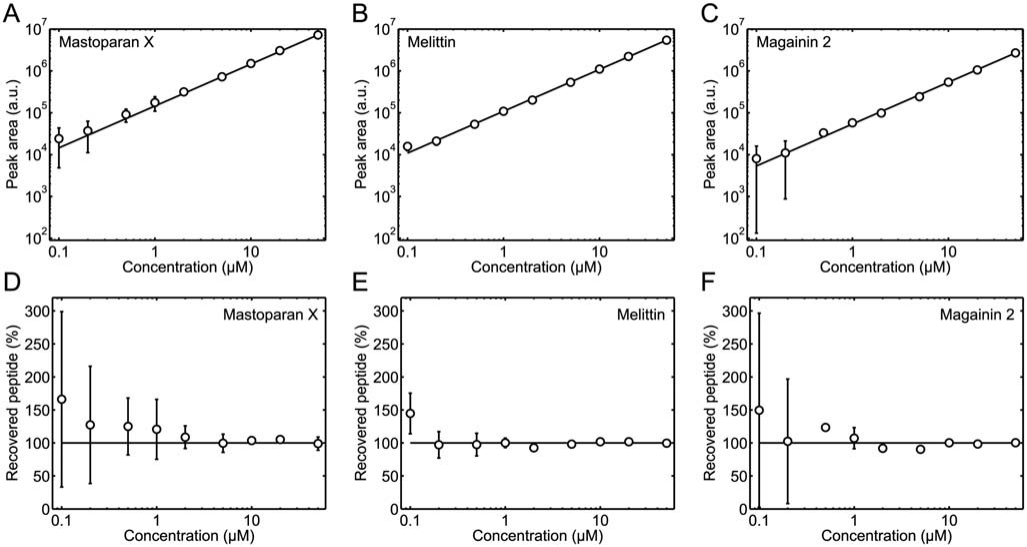
Peptide concentration standard curves. (*A*, *B*, and *C*) Peptide peak area as a function of the peptide concentration of mastoparan X (*A*), melittin (*B*), and magainin 2 (*C*) standard solutions. The solid lines are the best least squares fits of Eq 1 to the data. (*D*, *E*, and *F*) Percentage of recovered peptide, as calculated by Eq 2, as a function of the peptide concentration of mastoparan X (*D*), melittin (*E*), and magainin 2 (*F*) standard solutions. The concentrations on the horizontal axes are, in all panels, the concentrations of the 200 μL standard solutions before 50 μL 5 mM POPC/POPG (3:1) LUV solutions were added to the standard solutions. The data are the average of three separate experiments. The error bars show the standard deviations. The error bars are not shown if they are smaller than the symbols. Linear concentration standard curves were obtained for all three peptides.

For the concentration standard curve measurements, a number of 200 μL peptide standard solutions were prepared directly in limited volume inserts in HPLC autosampler vials. Then, 50 μL 5 mM POPC/POPG (3:1) LUV solution was added to each of the limited volume inserts to prevent adsorption of peptides to the walls of the inserts. Thus, when these solutions were analyzed by analytical HPLC, both a peptide peak and a lipid peak were visible in the chromatograms, see Fig 1. Only the peptide peaks were used in the further data analysis: the peptide peaks were integrated and their areas plotted as a function of the peptide concentration of the 200 μL standard solutions, see Fig 2A, 2B, and 2C. The obtained concentration standard curves were then fitted with a straight line:

$$\begin{array}{c} A_{\mathrm{pep}}=S\times C_{\mathrm{pep}} \end{array}$$ (1)

where *A*_pep_ is the peptide peak area, *C*_pep_ is the peptide concentration of the 200 μL standard solutions, and *S* is the slope of the straight line. Good agreement between the experimental data and the linear fits was generally observed. However, it should be noted that there were some uncertainties in the peptide peak areas measured for the lowest peptide concentrations of the concentration standard curves, owing to these lowest peptide concentrations being close to the detection limit of the HPLC method.

Next, we considered another way to represent the peptide concentration standard curves in Fig 2A, 2B, and 2C; thus, the peptide peak areas of the standard curves, *A*_pep_, were recalculated to percentages of recovered peptide, *R*_pep_, using the equation

$$\begin{array}{c} R_{\mathrm{pep}}=\frac{A_{\mathrm{pep}}}{S\times C_{\mathrm{pep}}}. \end{array}$$ (2)

The percentage of recovered peptide as a function of the peptide concentration of the 200 μL standard solutions is shown in Fig 2D, 2E, and 2F. In general, the percentage of peptide recovered in the concentration standard curve measurements was close to 100%, as it should be. However, for the lowest peptide concentrations, large uncertainties in the percentage of recovered peptide were observed, underlining the conclusion from the data representation in Fig 2A, 2B, and 2C, that the lowest peptide concentrations of the concentration standard curves were determined with some uncertainty.

In the following sections, we present the results of a number of systematic adsorption experiments. Peptide solutions for these adsorption experiments were generally prepared and incubated in a given type of sample container under a given set of experimental conditions, specific for that adsorption experiment. Then, 200 μL of the peptide solutions were transferred by pipette to limited volume inserts in HPLC autosampler vials. Subsequently, 50 μL POPC/POPG (3:1) LUV was added to each of the limited volume inserts to prevent adsorption of peptides to the walls of the inserts. From the acquired HPLC chromatograms, the peptide peak areas were then determined. These peptide peak areas were recalculated to percentages of recovered peptide using Eq 2 where *S* is the slope of the standard curves in Fig 2 and *C*_pep_ is the expected peptide concentration of the 200 μL solutions that were transferred to the limited volume inserts. In cases where the percentage of recovered peptide was found to be < 100%, some of the peptide had been lost in the experimental process, either because peptides adsorbed onto the walls of the sample containers during incubation (Step 2 of the sample preparation protocol for adsorption experiments in the Materials and Methods section) or because peptides adsorbed to the pipette tips when 200 μL of the solutions were transferred to limited volume inserts in HPLC autosampler vials (Step 3 of the sample preparation protocol for adsorption experiments in the Materials and Methods section). The latter explanation was ruled out by a control experiment, see S1 Results. Thus, any loss of peptide reported in the following sections is ascribed to adsorption of peptide onto the walls of the sample containers during incubation in the containers before transfer to the limited volume inserts.

### Peptide loss during 1 h incubation in sample containers

**Fig 3:**
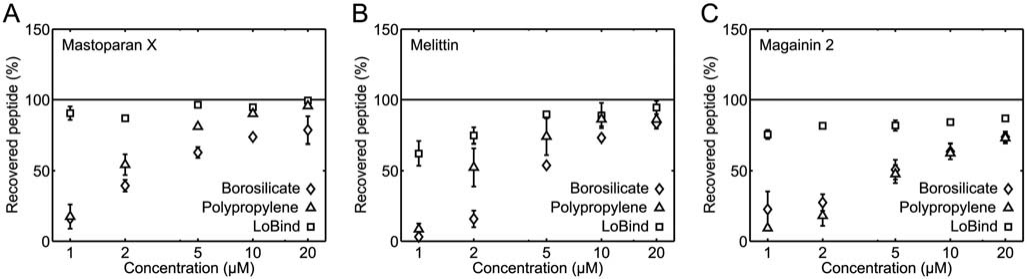
Peptide loss during 1 h incubation of 220 μ L solutions in sample containers. The percentage of recovered peptide was measured as a function of the peptide concentration for mastoparan X (*A*), melittin (*B*), and magainin 2 (*C*) solutions in borosilicate glass vials, polypropylene tubes, or Protein LoBind tubes. In all panels, the data are the average of two separate experiments. The error bars show the standard deviations. The error bars are not shown if they are smaller than the symbols. The data demonstrate that all three peptides tend to adsorb to the walls of the borosilicate glass vials and polypropylene tubes; at low peptide concentrations, only 10–20% of the expected peptide contents were recovered in these containers. In contrast, peptides do not absorb to Protein LoBind tubes to the same extent.

We started our adsorption experiments by measuring the loss of peptide in 220 μL solutions of varying peptide concentration incubated for 1 h in borosilicate glass vials or polypropylene tubes. For comparison, we also measured the loss of peptide in 220 μL solutions of varying peptide concentration incubated for 1 h in Protein LoBind tubes, which are tubes designed to minimize the surface adsorption of proteins and peptides. Fig 3 shows the results of these experiments. A number of common observations were done for all three investigated peptides. First, for 1 μM peptide solutions incubated in borosilicate glass vials or polypropylene tubes, only 10–20% peptide was recovered, meaning that most of the peptide adsorbed to the walls of these containers at this concentration. Second, for peptide solutions of higher concentrations incubated in borosilicate glass vials or polypropylene tubes, increasing percentages of peptide were recovered for increasing peptide concentrations, indicating that container walls became increasingly saturated with peptide for increasing peptide concentrations. Third, peptides were not lost to the same extent in the Protein LoBind tubes as in the borosilicate glass vials and polypropylene tubes.

**Fig 4:**
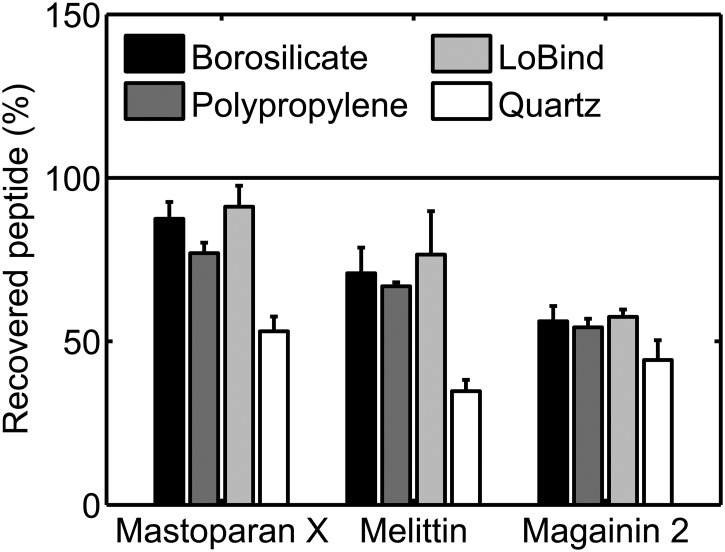
Peptide loss during 1 h incubation of 2 mL 2 μ M solutions in sample containers. The percentage of recovered peptide was measured for mastoparan X, melittin, and magainin 2 solutions in borosilicate glass vials, polypropylene tubes, Protein LoBind tubes, or quartz glass cuvettes. The data are the average of two separate experiments. The error bars show the standard deviations. The data demonstrate that peptide surface adsorption is not just a phenomenon that occurs at high surface area-to-solution volume ratios.

Next, we investigated the adsorption of peptide at a lower surface area-to-solution volume ratio. Specifically, we measured the loss of peptide in 2 mL 2 μM peptide solutions incubated for 1 h in borosilicate glass vials, polypropylene tubes, or Protein LoBind tubes. Additionally, we also measured the loss of peptide in 2 mL 2 μM peptide solutions incubated for 1 h in quartz glass cuvettes. The results of these experiments are shown in Fig 4. For peptide solutions incubated in borosilicate glass vials or polypropylene tubes, a higher percentage of peptide was generally recovered from the 2 mL 2 μM solutions than from the 220 μL 2 μM solutions (compare Figs 3 and 4), albeit significant surface adsorption still occurred in some cases in the 2 mL solutions. For mastoparan X and melittin solutions incubated in Protein LoBind tubes, similar percentages of peptide was recovered from the 2 mL 2 μM and the 220 μL 2 μM solutions. For magainin 2 solutions incubated in Protein LoBind tubes, a lower percentage of peptide was recovered in the case of 2 mL 2 μM solutions than in the case of 220 μL 2 μM solutions. For 2 mL 2 μM peptide solutions incubated in quartz glass cuvettes, peptides adsorbed rather effectively to walls of the cuvettes and/or the magnetic stirring bar: the percentage of peptide recovered in the quartz glass cuvettes was ≤ 50% for all three peptides.

### Peptide loss during successive transfers between sample containers

**Fig 5:**
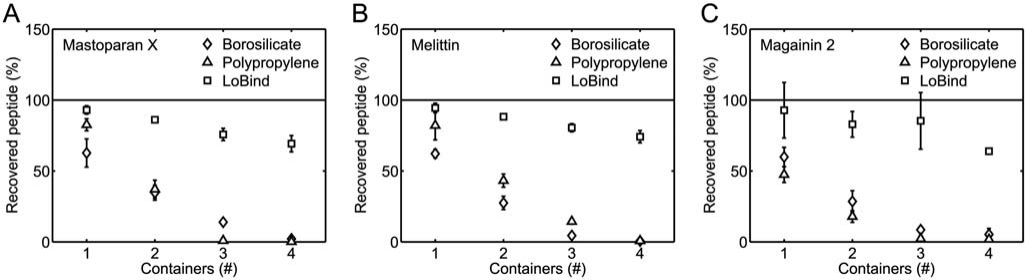
Peptide loss during successive transfers of 250 μ L 5 μ M solutions between sample containers. The solutions were successively transferred between borosilicate glass vials, polypropylene tubes, or Protein LoBind tubes. The percentage of recovered peptide was measured for mastoparan X (*A*), melittin (*B*), and magainin 2 (*C*) solutions as a function of the number of sample containers in which the solutions had been incubated. In all panels, the data are the average of two separate experiments. The error bars show the standard deviations. The error bars are not shown if they are smaller than the symbols. The data show that peptide is dramatically lost when peptide solutions are successively transferred between borosilicate glass vials or polypropylene tubes.

To further document the surface adsorption of the three investigated peptides, we successively transferred 250 μL 5 μM peptide solutions between 1–4 borosilicate glass vials, polypropylene tubes, or Protein LoBind tubes. Solutions were incubated for 1 h between each transfer step. Fig 5 shows the percentage of recovered peptide as a function of the number of containers in which the peptide solutions had been incubated. The data in the figure confirm the observation from Figs 3 and 4 that peptides adsorb onto the walls of borosilicate glass vials and polypropylene tubes; that is, in all cases where solutions had been transferred between 4 borosilicate glass vials or polypropylene tubes, only a small percentage of peptide, close to 0%, was recovered. When it comes to the Protein LoBind tubes, the data in Fig 5 is in agreement with the data in Fig 3: peptides are not lost to the same extent when peptide solutions are transferred successively between Protein LoBind tubes than when peptide solutions are transferred successively between borosilicate glass vials or polypropylene tubes.

### Effect of NaCl concentration on peptide loss

**Fig 6:**
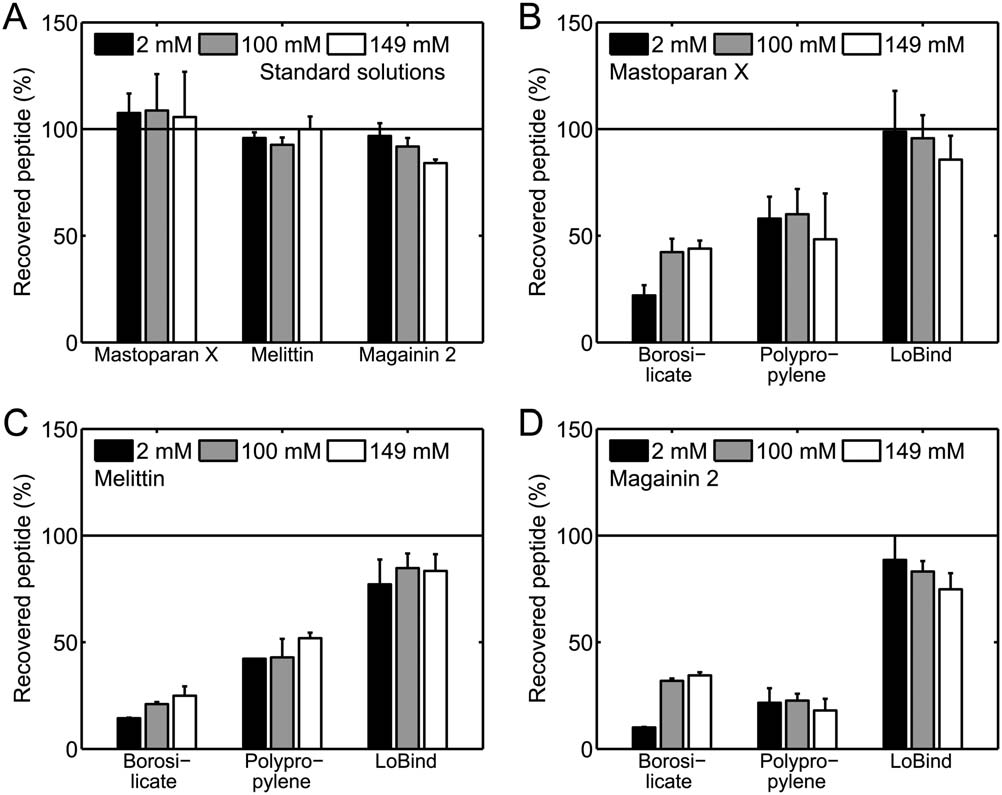
Effect of NaCl concentration on peptide loss in 220 μ L 2 μ M solutions. (*A*) Percentage of recovered peptide for 200 μL 2 μM standard solutions prepared in 10 mM HEPES buffers of varying NaCl concentration directly in limited volume inserts. (*B*, *C*, and *D*) Percentage of recovered peptide for 220 μL 2 μM mastoparan X (*B*), melittin (*C*), and magainin 2 (*D*) solutions incubated in 10 mM HEPES buffers of varying NaCl concentration for 1 h in borosilicate glass vials, polypropylene tubes, or Protein LoBind tubes. The data are the average of two separate experiments, except in (*A*) in which three experiments are averaged. The error bars show the standard deviations. The percentage of recovered peptide was not strongly influenced by the NaCl concentration.

So far, we have presented results of adsorption experiments carried out in 10 mM HEPES buffer with 100 mM NaCl. However, since it has previously been reported that the ionic strength of the buffer might impact the adsorption of cationic peptides to solid surfaces of borosilicate glass and polypropylene [11], we also carried out adsorption experiments in buffers of other NaCl concentrations than 100 mM. Before carrying out these adsorption experiments, we first needed to confirm that the accuracy of the HPLC method did not depend on the NaCl concentration of the buffer. Thus, we prepared a number of 200 μL 2 μM peptide standard solutions in 10 mM HEPES buffers with 2, 100, or 149 mM NaCl directly in limited volume inserts in the HPLC autosampler vials, similar to the way that the solutions for the concentration standard curves in Fig 2 were prepared directly in limited volume inserts. Fig 6A shows that approximately 100% peptide was recovered for all three peptides in these control experiments, regardless of the NaCl concentration, demonstrating that the HPLC method can be used to measure peptide concentrations in buffers of varying NaCl concentrations.

Then, we prepared a number of 220 μL 2 μM peptide solutions in borosilicate glass vials, polypropylene tubes, or Protein LoBind tubes using 10 mM HEPES buffers with 2, 100, or 149 mM NaCl. These solutions were incubated for 1 h. Fig 6B, 6C, and 6D, shows the percentage of peptide recovered from the solutions. For the solutions incubated in borosilicate glass vials, there might be a slight trend that the percentage of recovered peptide increases with the NaCl concentration. For the solutions incubated in polypropylene tubes and Protein LoBind tubes, we generally did not find that the NaCl concentration significantly affected the percentage of recovered peptide.

### Adsorption and desorption kinetics of mastoparan X

**Fig 7:**
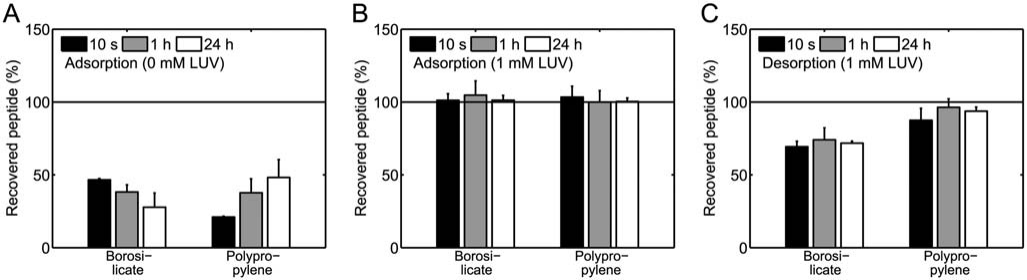
Adsorption and desorption kinetics of mastoparan X in 220 μ L 2 μ M solutions. The kinetics were investigated in borosilicate glass vials and polypropylene tubes. (*A*) Adsorption kinetics in buffer. (*B*) Adsorption kinetics in 1 mM POPC/POPG (3:1) LUV solution. (*C*) Desorption kinetics. The desorption from the container walls was induced by 1 mM POPC/POPG (3:1) LUV. The data are the average of two separate experiments. The error bars show the standard deviations. Generally, adsorption and desorption are fast processes that take place within a few seconds.

Next, we used mastoparan X as a model peptide to investigate the kinetics of peptide adsorption to the walls of the borosilicate glass vials and polypropylene tubes. To investigate the adsorption kinetics, 220 μL 2 μM mastoparan X solutions were incubated for 10 s, 1 h, or 24 h in borosilicate glass vials or polypropylene tubes. Fig 7A shows the recovered percentage of mastoparan X for the three different incubation times. The percentage of recovered peptide was found to be weakly dependent on the incubation time, showing that adsorption of mastoparan X to the walls of the borosilicate glass vials and the polypropylene tubes is a fast process that occurs during the first few seconds after addition of peptide to the solutions, probably while the solutions are still being vortexed.

We also studied the adsorption kinetics of mastoparan X in the presence of 1 mM POPC/POPG (3:1) LUV in the borosilicate glass vials and polypropylene tubes. For that purpose, 220 μL 2 μM mastoparan X solutions with 1 mM POPC/POPG (3:1) LUV were incubated for 10 s, 1 h, or 24 h in borosilicate glass vials or polypropylene tubes. Fig 7B shows the percentage of recovered peptide for the three different incubation times. For all three incubation times in both types of sample containers, the presence of 1 mM POPC/POPG (3:1) LUV prevents surface adsorption of mastoparan X, indicating that nearly 100% mastoparan X partitions onto the LUVs.

Finally, we also used mastoparan X as a model peptide to investigate the kinetics of peptide desorption from the walls of the borosilicate glass vials and polypropylene tubes. For that purpose, we used 1 mM POPC/POPG (3:1) LUV to induce desorption in 220 μL 2 μM mastoparan X solutions in borosilicate glass vials or polypropylene tubes. More specifically, we first prepared solutions of mastoparan X, and then we added 1 mM POPC/POPG (3:1) LUV to these solutions. After addition of LUV, we incubated the solutions for 10 s, 1 h, or 24 h. Fig 7C shows the percentage of recovered peptide for the three different incubation times. The percentage of recovered peptide was found to be largely independent on the incubation time; thus, similarly to the adsorption process, we found that the desorption process was a fast process that happened during the vortexing within the first few seconds after LUV addition. In the case of the borosilicate glass vials, the LUVs did not induce complete desorption of mastoparan X. Since the data in Fig 7B indicate that nearly 100% of the peptide was partitioned onto the LUVs, the observation on incomplete desorption could indicate that mastoparan X was partly irreversibly adsorbed to the walls of the borosilicate glass vials. In this context, it should be mentioned that not even longer periods of vortexing induced any further desorption of mastoparan X from the walls of the borosilicate glass vials.

## Discussion

Adsorption to the walls of standard laboratory glassware and plasticware have been reported for many different types of proteins and peptides [16–18]. In this article, we have been concerned with adsorption to glassware and plasticware of one specific type of peptides, namely, cationic membrane-active peptides. Specifically, we studied the surface adsorption of the three cationic membrane-active peptides mastoparan X, melittin, and maganin 2 to borosilicate glass vials, polypropylene tubes, and Protein LoBind tubes. We found that, at typical experimental peptide concentrations, a significant amount of peptide might be lost from solution due to rapid adsorption to the walls of the sample containers. This fact raises the question of how cationic membrane-active peptides optimally should be handled to reduce their adsorption to sample container walls. Based on the data presented in this article as well as information from the literature, we discuss this question in the following paragraphs.

### Choice of sample containers

We generally observed more surface adsorption of the three investigated peptides in the borosilicate glass vials and polypropylene tubes than in the Protein LoBind tubes (Figs 3, 5 and 6). This observation suggests that the Protein LoBind tubes, and possibly also other commercially available low-adsorption tubes [10, 19], can be a useful tool for minimizing the surface adsorption of cationic membrane-active peptides.

### Peptide concentration

We found that relatively more peptide adsorbs to the borosilicate glass vials and polypropylene tubes at low peptide concentrations than at high peptide concentrations (Fig 3). This observation indicates that the walls of these containers become saturated with peptide at high peptide concentrations. Accordingly, our results suggest that it might be beneficial to use a high peptide concentration when handling cationic membrane-active peptides, at least when keeping peptide solutions in borosilicate glass and polypropylene sample containers.

### Surface-to-volume ratio

For peptide solutions incubated in the borosilicate glass vials and polypropylene tubes, we found that relatively less peptide adsorbs to the container walls at low surface area-to-solution volume ratios than at high ratios (compare Figs 3 and 4). This finding indicates that at low surface area-to-solution volume ratios the equilibrium is shifted toward the aqueous phase and/or the container walls become increasingly saturated with peptide. Consequently, it might be advantageous to keep the sample volume-to-container surface ratio high when handling cationic membrane-active peptides, at least when keeping peptide solutions in borosilicate glass and polypropylene sample containers.

### Pretreatment of containers with peptide

It has previously been reported that the surface adsorption of mastoparan X, melittin, and magainin 2a to various types of sample containers can be reduced if the containers have been presaturated with solutions containing one of these respective peptides [6–8]. However, the results presented in this article indicate that caution should be taken when following this strategy, at least when working in borosilicate glass and polypropylene sample containers. More specifically, using mastoparan X as a model peptide, we demonstrated that adsorption of cationic membrane-active peptides to borosilicate glass vials and polypropylene tubes may be, at least partly, reversible (Fig 7). Accordingly, there is a risk that the walls of presaturated sample containers could serve as a reservoir of peptides that might be released into solution in response to some specific experimental step, such as addition of lipid vesicles to the containers, leading to a higher than expected experimental peptide concentration.

### Ionic strength

It has previously been reported that the ionic strength of the buffer impacts the adsorption of the cationic peptide salmon calcitonin to solid surfaces of borosilicate glass and polypropylene [11]. Specifically, it was found that the adsorption of salmon calcitonin to borosilicate glass is lowest at high ionic strengths whereas the adsorption to polypropylene is lowest at low ionic strengths. In the present article, we found that when solutions of mastoparan X, melittin, or magainin 2 are incubated in borosilicate glass vials there might be a slight trend that the percentage of recovered peptide increases with the NaCl concentration of the buffer, but there are no indications that the NaCl concentration can be adjusted to significantly alleviate the surface adsorption (Fig 6). For similar peptide solutions incubated in polypropylene tubes, we observed no clear signs that the percentage of recovered peptide depends on the NaCl concentration of the buffer. Thus, our results do not indicate that the ionic strength of the buffer can be tuned to prevent the surface adsorption of cationic membrane-active peptides to borosilicate glass and polypropylene sample containers.

### Lipids

We found that the presence of 1 mM POPC/POPG (3:1) LUV in the buffer prevents the adsorption of the three investigated peptides to borosilicate glass and polypropylene surfaces. (This is demonstrated directly in Fig 7 and indirectly by the fact that the LUVs are required in the borosilicate glass limited volume inserts in the HPLC autosampler vials to create the linear standard curves in Fig 2.) Likely, the peptides partition onto the LUVs instead of adsorbing to the container walls. This is an interesting point inasmuch as experiments with cationic membrane-active peptides often are carried out in samples containing lipid membranes, either synthetic lipid membranes or cellular membranes [20]. Thus, it might be possible to design these experiments in a way that leads to minimal adsorption of the peptides to the container walls, for example, by keeping the lipid concentration high and/or by selecting lipids for which the cationic membrane-active peptides have a high affinity. In this context, it should be mentioned that cationic membrane-active peptides generally have a much higher affinity for anionic lipids, such as POPG, than for zwitterionic lipids, such as POPC [21–23]. Accordingly, anionic lipids might be more effective than zwitterionic lipids at preventing surface adsorption of cationic membrane-active peptides to sample container walls.

### Surfactants

The presence of surfactants has been reported to reduce the adsorption of cationic membrane-active peptides to solid surfaces of glass and plastic. For example, it has been reported that the adsorption of salmon calcitonin to borosilicate glass and polypropylene could be reduced if certain types of surfactants are present in the solution [11]. Along these lines, we have in our lab obtained initial data that indicate that the presence of the anionic surfactant sodium dodecyl sulfate in solution prevents the adsorption of mastoparan X, melittin, and magainin 2 to the walls of the borosilicate glass limited volume inserts used in the HPLC experiments (data now shown). Generally, the surfactants might reduce the peptide surface adsorption either by blocking the container walls and/or by increasing the propensity of the peptides to stay in the aqueous solution [11]. However, even though surfactants might effectively reduce the surface adsorption of cationic membrane-active peptides, there are many experimental situations in which the presence of surfactants might be problematic. In particular, as surfactants are capable of interacting with and disrupting lipid membranes [24], caution should be taken when employing surfactants as a remedy for reducing the surface adsorption of cationic membrane-active peptides in experiments with lipid membranes.

### Pretreatment of containers with cationic polymer

Finally, it should be mentioned that adsorption of the cationic polymer poly(ethylenimine) to the walls of quartz cuvettes was reported to prevent the surface adsorption of the cationic membrane-active peptide penetratin [9]. This approach provides a potential strategy to reduce the surface adsorption for investigators working with cationic membrane-active peptides in quartz glass cuvettes and possibly also in other types of sample containers. However, as a general remark, if this strategy is pursued, thorough care should be taken that the polymer does not desorb from the container walls or in other ways interfere with the experimental system of interest.

## Conclusion

In this article, we systematically and quantitatively characterized the adsorption of the three cationic membrane-active peptides mastoparan X, melittin, and magainin 2 to standard laboratory glassware and plasticware. Our results show that, at typical experimental peptide concentrations, an overwhelming amount of peptide might be lost from solution due to rapid adsorption to the walls of the glassware and plasticware. Accordingly, our results emphasize that it is important that investigators working with cationic membrane-active peptides keep these adsorption effects in mind when designing and conducting their experiments.

## Supporting Information

S1 Results Adsorption of peptide on pipette tips. The document presents experiments that demonstrate that the loss of peptide reported in this article is not due to adsorption of peptide to the pipette tips.
(PDF)

S1 Dataset Peptide peak areas determined by analytical HPLC. The dataset contains the peptide peak areas used to prepare Figs 2–7 and the figure in S1 Results.
(XLSX)
