# Supplementary material for: Adsorption of Cationic Peptides to Solid Surfaces of Glass and Plastic
Source: PLoS One. 2015 May 1;10(5):e0122419. doi: 10.1371/journal.pone.0122419 (PMC4416745; doi:10.1371/journal.pone.0122419)
Supplement: S1 Results — The document presents experiments that demonstrate that the loss of peptide reported in this article is not due to adsorption of peptide to the pipette tips. (PDF) [file pone.0122419.s001.pdf]

S1 Results

Adsorption of cationic peptides to solid surfaces of glass and plastic

Kasper Kristensen,[1,3] Jonas R. Henriksen,[2,3] and Thomas L. Andresen[1,3]
[1]Department of Micro- and Nanotechnology, DTU Nanotech, Technical University of Denmark, Kongens Lyngby, Denmark, [2]Department of Chemistry, DTU Chemistry, Technical University of Denmark, Kongens Lyngby, Denmark, [3]Center for Nanomedicine and Theranostics, Technical University of Denmark, Kongens Lyngby, Denmark

Adsorption on pipette tips

Method

Steps 1 to 3 of the protocol for this adsorption experiment was as follows:

1. Peptide solutions of 1 µM were prepared in Protein LoBind tubes by adding 2.2 µL 100 µM peptide stock solution to 217.8 µL buffer to a final volume of 220 µL.
2. Immediately after addition of peptide, the solutions were vigorously vortexed for a few seconds and then incubated for 1 h. After this incubation time, 200 µL of each of the solutions was aspirated into a pipette tip and kept in the pipette tip for $\sim$ 5 s. Then, each of the 200 µL solutions in the pipette tips were deposited back into the same Protein LoBind tube from which they were aspirated. Pipetting manipulations were repeated 1-4 times for each solution using new pipette tips for each manipulation.
3. After the last pipetting manipulation, the 200 µL solutions in the pipette tips were not deposited back into the Protein LoBind tubes but instead into limited volume inserts in HPLC autosampler vials.

The rest of the protocol was identical to the general sample preparation protocol for the adsorption experiments, described in the main article.

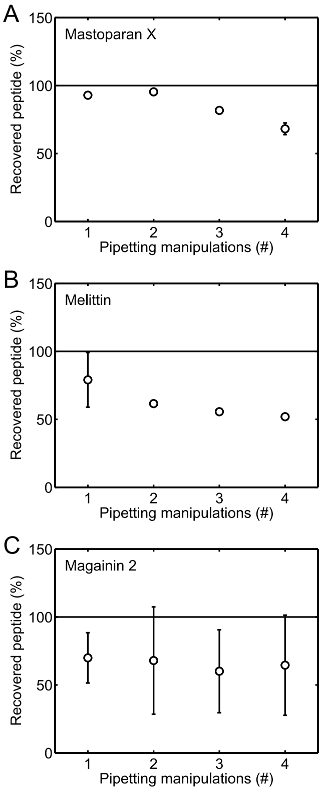

Adsorption loss during pipetting of 200 µL 1 µM peptide solutions. The solutions were subject to 1-4 pipetting manipulations. The percentage of recovered peptide was measured for mastoparan X (*A*), melittin (*B*), and magainin 2 (*C*) solutions as a function of the number of pipetting manipulations. The data are the average of two separate experiments. The error bars show the standard deviations. The error bars are not shown if they are smaller than the symbols. Only a small loss of peptide, if any at all, occurs during pipetting.

Results

The figure in the present document shows that the percentage of recovered peptide only decreases weakly as a function of the number of pipetting manipulations. Thus, we conclude that in our experimental setup aspiration of 200 µL peptide solutions into the pipette tips only entails a negligible loss of peptide due to adsorption on the pipette tips. Consequently, the loss of peptide in the adsorption experiments presented in the main article must be due to adsorption of peptide to the walls of the sample containers used in the adsorption experiments.

It should be emphasized that the conclusion that peptide adsorption on pipette tips is low should not be generalized, that is, our results only show that adsorption on pipette tips is low in our specific experimental setup. Our results do not guarantee that adsorption on pipette tips will not be high with other peptides and/or with other types of pipette tips and/or under other experimental conditions.
